# PCB Exposure and *in Vivo* CYP1A2 Activity among Native Americans

**DOI:** 10.1289/ehp.7370

**Published:** 2004-12-09

**Authors:** Edward F. Fitzgerald, Syni-An Hwang, George Lambert, Marta Gomez, Alice Tarbell

**Affiliations:** ^1^University at Albany, School of Public Health, Rensselaer, New York, USA; ^2^New York State Department of Health, Center for Environmental Health, Troy, New York, USA; ^3^University of Medicine and Dentistry of New Jersey, Robert Wood Johnson Medical School, New Brunswick, New Jersey, USA; ^4^Akwesasne Task Force on the Environment, Mohawk Nation at Akwesasne, Hogansburg, New York, USA

**Keywords:** cytochrome P-450 1A2, hazardous waste, Indians, North American, PCB, polychlori-nated biphenyls

## Abstract

Cytochrome P-450 1A2 (CYP1A2) is an enzyme involved in the metabolic activation of some carcinogens and is believed to be induced by xenobiotics. Very few studies, however, have investigated the association between environmental exposures and *in vivo* CYP1A2 activity in humans. To address this issue, a study was conducted of CYP1A2 activity among Native Americans exposed to polychlorinated biphenyls (PCBs) from the consumption of fish from the St. Lawrence River. At the Mohawk Nation at Akwesasne (in New York and in Ontario and Quebec, Canada), 103 adults were interviewed, and they donated blood for serum PCB analysis and underwent the caffeine breath test (CBT), a safe and noninvasive procedure that uses caffeine as a probe for CYP1A2 activity *in vivo*. The results supported the findings of other studies that CBT values are higher among smokers and men and lower among women who use oral contraceptives. Despite a relatively low average total PCB body burden in this population, the sum of serum levels for nine mono- or di-*ortho*-substituted PCB congeners showed positive associations with CBT values (*p* = 0.052 wet weight and *p* = 0.029 lipid adjusted), as did toxic equivalent quantities (TEQs; *p* = 0.091 for wet weight and 0.048 for lipid adjusted). Regarding individual congeners, serum levels of PCB-153, PCB-170, and PCB-180 were significantly correlated with CBT values. The results support the notion that CYP1A2 activity may be a marker of an early biological effect of exposure to PCBs in humans and that the CBT may be a useful tool to monitor such effects.

Cytochrome P-450 1A2 (CYP1A2) is a member of the cytochrome P-450 superfamily of isozymes. It is involved in the metabolic activation of several carcinogens such as aromatic and heterocyclic amines, nitrosamines, and mycotoxins (Eaton et al. 1995). In humans, CYP1A2 has been detected primarily in the liver, in contrast to the closely related CYP1A1, which is expressed in extrahepatic tissues such as lung, placenta, and lymphocytes (Kawajiri 1999). The induction of CYP1A2 has been reported as a consequence of cigarette smoking (Kalow and Tang 1991a; Kotake et al. 1982), the consumption of certain foodstuffs such as charbroiled meat (Conney et al. 1976) and cruciferous vegetables (Pantuck et al. 1979; Vistisen et al. 1992), and therapeutic drugs such as rifamin (Wietholtz et al. 1995), carbamazepine (Parker et al. 1998), and omeprazole (Rost et al. 1994). There is wide variability in CYP1A2 expression among individuals in most ethnic and racial groups studied (Landi et al. 1999), but other than a rare point mutation detected among the Chinese (Huang et al. 1999), no genetic polymorphisms for CYP1A2 have been identified (Nakajima et al. 1994).

Like CYP1A1, CYP1A2 is thought to be induced by exposure to xenobiotics such as 2,3,7,8-tetrachlorodibenzo-*p*-dioxin (TCDD), polychlorinated biphenyls (PCBs), and other structurally related chemicals that have the ability to bind to the aryl hydrocarbon (Ah) receptor. The evidence for this belief is derived primarily from animal models (Safe 1994) or *in vitro* studies of mRNA or enzyme levels in treated human liver cells (Lake et al. 1996; Zeiger et al. 2001). Very few studies have investigated *in vivo* CYP1A2 activity in humans exposed to TCDD, PCBs, or related chemicals, and the results have been contradictory. For example, Lambert et al. (1990) found that persons exposed to polybrominated biphenyls (PBBs) in Michigan had higher levels of CYP1A2-dependent caffeine metabolism than did unexposed controls, but no significant associations were observed between caffeine metabolism and serum TCDD levels among exposed chemical workers (Halperin et al. 1995). The present study addresses the question of whether PCB exposure has affected *in vivo* CYP1A2 activity among Mohawk men and women at Akwesasne.

Akwesasne is a Native American community of > 10,000 persons located along the St. Lawrence River in New York and in Ontario and Quebec, Canada (Figure 1). Less than 100 ft to the west of Akwesasne is the General Motors–Central Foundry Division Superfund hazardous waste site. This facility used Aroclor 1248, a commercial mixture of various PCB congeners, as a hydraulic fluid in its die-casting machines from 1959 to 1974 (Lacetti 1993). When these machines leaked, the fluids were collected in the wastewater system and disposed of on the property. In the past, concentrations of PCBs have ranged up to 40,000 ppm in on-site soils and sludges, and up to 5,700 ppm offshore in St. Lawrence River sediment (RMT 1986). The Aluminum Company of America (ALCOA) operates two aluminum-processing facilities in the area (ALCOA Plant West and ALCOA Plant East, the latter being formerly operated by Reynolds Metals, Inc.) and also has used Aroclor 1248 in its heat-transfer equipment. These facilities also have released PCBs into the St. Lawrence and its tributaries (Ecology and Environment Inc. 1992). The PCBs have entered the local food chain, with some species of local fish, reptiles, amphibians, birds, and mammals having levels that exceed the U.S. Food and Drug Administration’s tolerance limits for human consumption of 2 ppm (wet weight) for local fish and 3 ppm (lipid weight) for poultry (Skinner 1992; Sloan and Jock 1990). Dredging of the St. Lawrence River offshore from the General Motors facility has been completed, but remedial alternatives for the St. Lawrence River offshore from Reynolds and for the Grasse River near ALCOA are still being evaluated. On-site remediation is complete at Reynolds and ALCOA, but plans for remedial work at some on-site locations at General Motors await final review and approval.

The pollution is a major concern of the Mohawk people because their tradition and culture emphasize the interdependence of humans and the environment and because many residents formerly depended on local fish, waterfowl, and mammals for food. Previous articles described local fish consumption patterns among 139 Mohawk men (Fitzgerald et al. 1996, 1999) and 111 pregnant Mohawk women (Fitzgerald et al. 2004) and their association with serum PCB levels. In these reports we noted a 3-fold decline in the average rate of local fish consumption among both men and women in the past year relative to > 2 years prior. Such changes may be related to the advisories that have been issued over the past decade by Mohawk, state, and provincial authorities against the consumption of contaminated local fish [New York State Department of Environmental Conservation (NYSDEC) 2003; New York State Department of Health (NYSDOH) 2002]. The geometric mean serum PCB concentration was 2.8 ppb for men and 1.2 ppb for women. In both cases, a significant correlation was observed between estimated cumulative lifetime exposure to PCBs from local fish consumption and serum PCB levels.

In the earlier investigations (Fitzgerald et al. 1996, 1999, 2004), we used serum PCB concentrations as a marker of internal dose, whereas in this study we expanded the focus to include a measure of an early biological effect of exposure. More specifically, we tested the hypothesis that serum PCB levels are positively correlated with CYP1A2 activity among Mohawk men and women. We used a breath test that uses caffeine as a metabolic probe to safely and noninvasively monitor CYP1A2 activity *in vivo* (Lambert et al. 1983). The project was a collaborative effort among the New York State Department of Health, the St. Regis Mohawk Tribe, the Mohawk Council of Akwesasne, the Akwesasne Task Force on the Environment, and the State University of New York at Albany.

## Materials and Methods

### Ascertainment and interview.

Detailed descriptions of ascertainment and interview methods are published elsewhere (Fitzgerald et al. 1996, 1999, 2004). Briefly, 111 Mohawk women living at Akwesasne who became pregnant between 1 April 1992 and 31 March 1995 were identified through prenatal care clinics and other sources, interviewed in the second trimester, and asked to donate a nonfasting 20 mL venous blood sample for PCB analysis. The participation rate was 79%. Similarly, 139 adult Mohawk men, who were the husbands or close relatives of the women, also took part (participation rate of 69%). Both the men and women were asked about demographic characteristics; height and weight; use of medications; occupational, residential, and reproductive histories; recreational pursuits (e.g., gardening, swimming, fishing); alcohol and cigarette use; drinking-water source; and diet, emphasizing local foods such as fish, wildlife, meat, dairy products, and fruits and vegetables.

### Serum PCB analysis.

The blood was allowed to clot and then centrifuged to provide 10 mL serum. The chemical analysis was performed using methods (including quality assurance and control, accuracy, and precision) published elsewhere (Bush et al. 1982, 1984). Briefly, approximately 10 mL of serum was extracted using methanol, diethyl ether, and hexane and then transferred to a Florisil cleanup column containing 10 g of 4% deactivated Florisil topped with 1 cm anhydrous sodium sulfate. The eluate was evaporated to 1 mL and analyzed with a Hewlett-Packard 5890 gas chromatograph (Hewlett-Packard, Houston, TX) using a phenylmethyloctadecyl silyl-bonded fused-silica capillary column and an electron-capture detector. A computerized data management system reported each of 68 PCB-containing zones or peaks and summed the congener concentrations to report total PCBs. In some cases, the capillary column was unable to resolve two or more congeners, so the result was reported as a mixed peak.

The method detection limit (MDL) for the 68 congeners in serum ranged from 0.01 to 0.10 ppb, with a median MDL of 0.02 ppb per congener. However, values less than the MDL were reported by the laboratory and included in the statistical analysis. This decision was based on the fact that many chemists and statisticians believe that a reported result, even if it is below the “criterion for detection,” remains the best available estimate of the true value and is preferable to assigning an arbitrary constant such as one-half the detection limit in the statistical analysis [American Society for Testing and Materials (ASTM) 1989].

In addition to the determination of PCB congeners, the lipid content of the serum samples was also measured gravimetrically. However, given the relatively low lipid content of serum, this method may be prone to error. Consequently, serum cholesterol and triglycerides were measured enzymatically, and total lipids were calculated using the Centers for Disease Control and Prevention formula (Phillips et al. 1989). This change was implemented midway through the study period, so unfortunately these data were available for only 46 of the 103 participants.

### Caffeine breath test.

Medical histories were taken to identify those with a history of heart disease, stroke, seizure disorders, uncontrolled hypertension, arrhythmia, hepatitis, jaundice or other types of liver disease, adverse reaction to caffeine, or chemotherapy within the past 5 years that would preclude their participation in the caffeine breath test (CBT). Those currently taking prescription medications other than oral contraceptives and women who were currently breast-feeding also were excluded. Participants were asked to refrain from caffeine consumption for 24 hr and to fast for 8 hr before the CBT. Height and weight were measured, and blood pressure and heart rate were taken to ensure normal readings before the CBT was administered. Each participant signed an informed consent and was compensated $30 for his or her time and effort. The women were tested after they delivered to avoid any potential risk of the caffeine to the fetus.

The substrate was synthesized (3-^13^C-methyl)caffeine (Kotake et al. 1982). The labeled caffeine dose, 3 mg/kg up to a maximum of 200 mg, was dissolved in 20 mL sterile water and ingested by the participants, followed by ingestion of a 20-mL water wash of the container. A 20-mL breath sample of expired air was collected immediately before and after the ingestion of the labeled caffeine at 30- and 60-min intervals, and stored in a Vacutainer. Pharmacologically, the labeled caffeine is rapidly absorbed and transported to the liver, where it is metabolized through 3-*N*-demethylation (Kotake et al. 1982). It then traverses to the one carbon pool and is exhaled in the breath as labeled CO_2_. The ^13^CO_2_:^12^CO_2_ ratio is determined by differential gas-isotope ratio mass spectrometry (Schoeller and Klein 1979). The excess ^13^C is calculated from the ratio found in the breath sample just before and after ingestion of the substrate and expressed as the dose exhaled per hour. Because 3-*N*-demethylation is catalyzed by CYP1A2, caffeine metabolism reflects hepatic CYP1A2 activity (Landi et al. 1999).

### Statistical analysis.

We used multiple linear regression analysis to test for the association between serum PCB concentrations and the CBT values, after controlling for significant background variables that could potentially confound any such association. Potential confounders consisted of a set of background variables [age, sex, body mass index (BMI), cigarette smoking, alcohol consumption, coffee consumption, occupational and recreational exposures to chemicals, medical conditions, and the use of illicit, prescription, and over-the-counter drugs], some of which have been related to CBT values in other studies (Horn et al. 1995; Kalow and Tang 1991b; Rost et al. 1994; Vistisen et al. 1992). As an initial screen, bivariate analyses were conducted to identify variables that were associated with the CBT values at *p* < 0.20. These variables were then regressed on the CBT values, and backward elimination was used to delete one at a time those that were associated at *p* > 0.10. The serum PCB concentrations were then added to the model to estimate the strength of their association with the CBT values after adjustment for all remaining background variables. Both the serum PCB concentrations and CBT values were log-transformed to normalize their distributions and stabilize their variances. The inclusion of the background variables in the final regression model was confirmed by determining whether the parameter estimates for the exposure variables changed by ≥10% when the background variables were deleted (Rothman and Greenland 1998).

The serum PCB levels were added to the regression models in four ways. First, the sum of concentrations for all 68 congeners was entered as total PCBs. Second, the sum of concentrations for the 3 congeners that are mono-*ortho*-substituted [International Union of Pure and Applied Chemistry (IUPAC) congeners 105, 118, 167] and the 6 that are di-*ortho*-substituted (IUPAC congeners 138, 153, 158, 170, 180, 194) derivatives of non-*ortho*-substituted PCBs (∑PCBs) were entered. Because the laboratory was not able to measure the non-*ortho*-substituted congeners themselves, the 9 congeners listed above (∑PCBs) represented the subset of the 68 congeners analyzed with the greatest affinity for the Ah receptor and consequently those that were most likely to induce CYP1A2 activity. Toxic equivalents (TEQs) were also computed for these nine congeners, using the World Health Organization (Van den Berg et al. 1998) toxic equivalency factors (TEFs), and then added to the regression models. Finally, individual regression models were also fitted for individual congeners. However, to prevent misconceptions regarding the level of certainty attached to the results, these congeners were limited to the five that had a median or mean concentration that equaled or exceeded their individual MDL. Additionally, the regressions were performed using the serum PCB concentrations of the subset of 46 participants with cholesterol and trigylceride determinations after expressing their results as nanograms per gram of lipid.

## Results

Of the 250 men and women in the parent studies, 172 (68.8%) agreed to undergo the CBT. Of this number, 69 (40.1%) were determined to be ineligible, yielding a final sample size of 103. Selected characteristics of these participants are described in Table 1. The mean age was 28 years, with a range of 15–67 years. Sixty-two percent were male. The median percent caffeine dose exhaled in an hour was 1.6%, with a range of 0.1–6.1%. Approximately one-half had smoked cigarettes in the past 2 years. Forty-three percent of the women had taken oral contraceptives during the past 6 months.

The median serum total PCB level was 1.86 ppb (wet weight), with a maximum of 14.91 ppb (Table 2). The median serum concentrations for the nine mono- or di-*ortho*-substituted congeners ranged from non-detectable to 0.28 ppb, and their median sum was 0.83 ppb. The median TEQ for these congeners was 0.01 ppt. The most commonly detected congener was PCB-153, with 95% of all the samples having a concentration that exceeded the MDL.

The bivariate analyses revealed that smokers, men, older persons, those with lower body mass indices, those without a history of hypertension, current coffee drinkers, and those who did not take antibiotics had higher median CBT values at *p* < 0.20. Only smoking and sex, however, were associated with CBT values at *p* < 0.10 in the multiple regression analysis and affected the parameter estimates for serum PCB by ≥10% (Table 3). Consequently, those two factors were included in the final regression models. Among women, oral contraceptive use was associated with lower CBT values (*p* = 0.006) after adjustment for cigarette smoking. However, this variable could not be included in the final multiple regression analysis of CBT values on serum PCB concentrations because this analysis included both sexes and oral contraceptive use was restricted to women. It is important to note, however, that the median CBT value of 1.68 for women who did not use oral contraceptives was identical to that for men, and that the results of a regression analysis limited to women and controlling for oral contraceptive use were similar to those of the final models for both sexes combined (data not shown).

Table 4 gives the results of the multiple regression analysis of CBT values on serum concentrations (both wet weight and lipid adjusted) of total PCBs, the ∑PCBs, and TEQs. It also displays the findings for the five individual mono- or di-*ortho*-substituted congeners that had median or mean serum concentrations that exceeded their respective MDLs. After adjusting for cigarette smoking and sex, serum total PCB was not associated with CBT values in either the wet-weight or lipid-adjusted analysis. However, the sum of the mono- and di-*ortho*-substituted congeners was significantly related to CBT values in both analyses. Positive associations were also observed for PCB-153 (*p* = 0.045 for wet weight, *p* = 0.011 for lipid adjusted), PCB-170 (*p* = 0.079 for wet weight, *p* = 0.010 for lipid adjusted), and PCB-180 (*p* = 0.086 for wet weight, *p* = 0.009 for lipid adjusted). TEQs were also positively associated with CBT values (*p* = 0.091 for wet weight, *p* = 0.028 for lipid adjusted).

## Discussion

The results confirmed other studies indicating that smoking, sex, and oral contraceptive use significantly impact CBT values. Smoking is a potent inducer of CYP1A2 (Kalow and Tang 1991a; Kotake et al. 1982), probably due to polycyclic aromatic hydrocarbons and other carcinogens found in tobacco smoke. Oral contraceptive use appears to inhibit CYP1A2 activity *in vivo* (Abernethy and Todd 1985; Campbell et al. 1987b; Rietveld et al. 1984), thereby delaying caffeine metabolism. Some investigators have attributed the higher CBT values of men relative to women to parity (Horn et al. 1995), but oral contraceptives may also be involved because women who did not use oral contraceptives had an average CBT value identical to men in the present study. Kalow and Tang (1991b) also reported no principal gender difference when women using oral contraceptives were excluded from their analysis.

It is not surprising that serum total PCB was not related to CBT values because most of the 68 congeners measured in this investigation are not known to induce CYP1A2. When the analysis was restricted to the ∑PCBs believed to have some affinity for the Ah receptor, however, significant positive associations were found. To estimate the dioxin-like activity of these congeners, we calculated TEQs. Although this approach has uncertainties because of possible nonadditive effects, differences in the shape of the dose–response curve, and species responsiveness (van den Berg et al. 1998), TEQs and CBT values were positively correlated. In fact, after lipid adjustment, this relationship was statistically significant, probably because, relative to the wet-weight analysis, the lipid adjustment controls for variability in PCB levels due to differences in lipid content of the nonfasting serum samples (Phillips et al. 1989).

When the results were restricted to only those congeners with median serum levels greater than their MDL, PCB-153, PCB-170, and PCB-180 were statistically significant in either the wet-weight or lipid-adjusted analysis. PCB-153 and PCB-180 are persistent congeners that, together with PCB-138, were found in the greatest concentration in Mohawk serum. These congeners typically are the most dominant in human tissue worldwide (Hansen 1999). PCB-170 is also persistent but usually found in lower concentrations than are PCB-153 and PCB-180. None of these three congeners is found in Aroclor 1248 (Frame et al. 1996), the commercial mixture used locally, and as such may reflect more general exposures, possibly from Lake Ontario and the St. Lawrence River (Bush et al. 1985).

PCB-153 and PCB-180 are generally considered phenobarbital-type inducers of cytochrome P-450 (Parkinson et al. 1981). They differ from non-*ortho*-substituted PCBs, which are 3-methylcholanthrene-type inducers, in that they are more likely to induce the CYP2A family of isozymes than the CYP1A family (Safe 1994). The other seven mono- and di-*ortho*-substituted congeners measured in the present study, including PCB-170, are mixed type and induce both families (McFarland and Clark 1989). The significant association between PCB-153 and PCB-180 and CBT values may reflect overlap in the ability of PCBs to induce P-450 enzymes, because phenobarbital-type inducers may also induce CYP1A to some extent and methylcholanthrene-type inducers may induce CYPA2 (Wolff and Toniolo 1995). Given the tendency of serum concentrations of individual congeners to be correlated (DeVoto et al. 1997; Gladen et al. 1999; Koopman-Esseboom et al. 1994), PCB-153 and PCB-180 may also be proxies for other congeners that were not measured, especially non-*ortho*-substituted congeners that have a high affinity for the Ah receptor. In fact, Longnecker et al. (2000) have reported Pearson correlation coefficients between PCB-153 and PCB-180 on the one hand and selected non-*ortho*-substituted PCBs on the other that range from 0.35 to 0.86.

It is important to note that these associations between serum PCB concentrations and CBT values were observed despite the relatively low average body burden of PCBs in this population. The median was 1.8 ppb, which is less than the general population value of 3.1 ppb reported by Patterson et al. (1994) during the same time period. This finding probably reflects the low current rate of local fish consumption among the Mohawks, a behavioral change that may be related to the fish advisories issued over the past decade by tribal, state, and provincial agencies (Fitzgerald et al. 1999, 2004). It is also uncertain whether Native Americans possess the polymorphisms that control induction. Such polymorphisms have not yet been identified, but in a study of CYP1A2 phenotypes from Australia, China, Japan, Italy, and the United States, Kadlubar (1994) found a wide variation in the metabolic proficiency for CYP1A2 within each country. Although Native peoples were not included, such results suggest that they, like other racial and ethnic subgroups, include at least a subset of inducible persons. Knowledge of which individuals were genetically capable of induction would have clearly strengthened the correlations observed in the present study.

Another limitation is the lack of information on the concentrations of non-*ortho*-substituted congeners in the serum of study participants. This issue is important because these congeners are those most likely to induce CYP1A2 *in vitro* or in animal models (Safe 1994). Unfortunately, our laboratory was unable to reliably quantify at the time that the study was conducted with the very low level of non-*ortho*-substituted congeners typically found in human serum. As noted previously by Longnecker et al. (2000), however, serum concentrations of individual congeners tend to be highly correlated, so persons who had higher levels of the congeners that were measured would likely have higher levels of non-*ortho*-substituted and any other congeners that were not determined. Similarly, no serum data were available for TCDD or other dioxins, which are the most potent inducers of CYP1A2 (Safe 1994). However, not only are levels of PCB congeners in human serum intercorrelated, but so are levels of PCBs and dioxins (Gladen et al. 1999; Koopman-Esseboom et al. 1994; Patterson et al. 1994); consequently, the former may also be a surrogate for the latter, at least when exposures are at background levels (Longnecker et al. 2000).

Despite these limitations, the general pattern of results between serum PCB concentrations and CBT values is generally consistent with at least one of the two other studies that, to date, have attempted to link xenobiotic exposure to human CYP1A2 activity *in vivo*. Specifically, Lambert et al. (1990) found that Michigan residents exposed to PBBs had a significantly higher median CBT value than did a control group of urban nonsmokers. They also observed a significant correlation between serum PBB levels above the detection limit and CBT values in the exposed group. In contrast, Halperin et al. (1995) found little evidence of an overall association between serum TCDD concentrations and CYP1A2 activity among occupationally exposed herbicide workers. The only suggestion of a relationship was when three of four categories of workers defined by increasing concentrations of serum TCDD showed a greater risk of having an elevated level of CYP1A2 activity relative to unexposed controls, but the results were not statistically significant. However, Halperin et al. (1995) used the caffeine metabolite ratio (CMR) to indicate *in vivo* CYP1A2 activity, not the CBT. The CMR measures caffeine metabolites in urine, metabolites that are dependent on different enzymes and pathways than the CBT (Campbell et al. 1987a). There is some uncertainty about what metabolites are most appropriate for the urinary ratio (Butler et al. 1992) and, although the comparative ordering of values is unaffected, the CMR underestimates the true magnitude of CYP1A2 activity (Kalow and Tang 1993), suggesting that it may be a less sensitive indicator than the CBT.

The results of the present study are also consistent with two other studies that, although they did not measure *in vivo* CYP1A2 activity, did monitor human placental CYP1A1 induction. Lagueux et al. (1999) found that CYP1A1 activity was elevated in the placental tissue of Inuit women living in northern Quebec who are exposed to PCBs and other organochlorine compounds from the consumption of marine mammals. Similarly, Lucier et al. (1987) reported that Yu-Cheng women exposed to PCBs and dibenzofurans had higher levels of placental CYP1A1 activity than an unexposed control group, and that such activity was inversely correlated with the birth weight of their offspring.

## Conclusion

In conclusion, the results of the present study support previous observations that smokers and men have higher levels of *in vivo* CYP1A2 activity than do nonsmokers and women and that oral contraceptive use inhibits CYP1A2 activity. It is one of the first investigations to report positive correlations between serum PCB concentrations and CYP1A2 activity, despite the relatively low PCB body burdens of this population and the lack of data on individual non-*ortho*-substituted PCBs or dioxins. While serum PCB concentrations serve as a marker of internal dose, CYP1A2 activity indicates an early biological effect of such exposure, at least among those persons genetically predisposed. Although the health implications in humans remain uncertain, CYP1A2 is involved in the metabolic activation of some carcinogens, and consequently, individual differences may reflect susceptibility to environmentally related cancer risk (Landi et al. 1999). The data support the notion that human exposure to PCBs may induce CYP1A2 activity and that the CBT is a useful tool to monitor such effects *in vivo*.

## Figures and Tables

**Figure 1:**
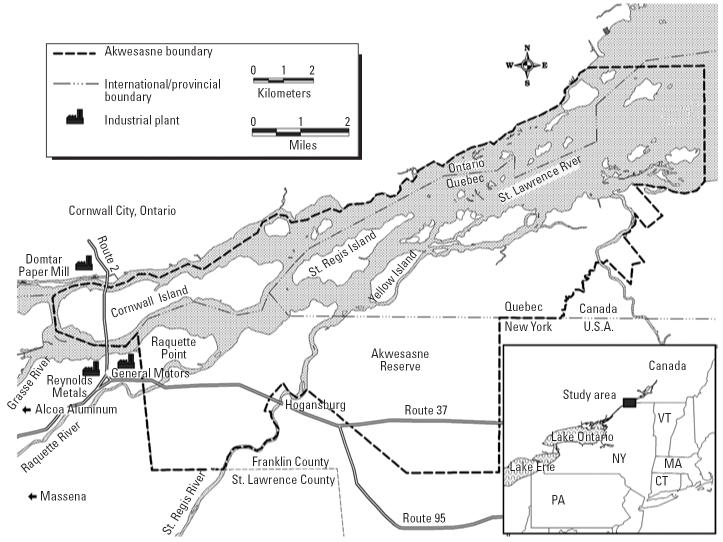
Map of the Mohawk Nation at Akwesasne.

**Table 1:** Selected characteristics of study participants (63 Mohawk men and 40 Mohawk women), Akwesasne, 1992–1995.

Characteristic*^a^*	Median	Mean ± SE	Range
Age (years)	28	30.3 ± 0.9	15–67
BMI (height/weight^2^)	26.4	27.1 ± 0.4	18.7–38.9
CBT (% dose)	1.6	1.8 ± 0.1	0.1–6.1
Cigarette smoking in past 2 years (% yes)	52.4		
Oral contraceptive use in past 6 months (women only, % yes)	42.9		
Current alcohol consumption (% yes)	55.0		
Current coffee consumption (% yes)	65.5		

a Sample size < 103 for some characteristics due to missing data.

**Table 2:** Serum PCB concentrations (ppb) in Mohawk men (*n* = 63) and women (*n* = 40), Akwesasne, 1992–1995.

Congener	Median	Mean ± SE	Range	Percent > MDL
PCB-105	< MDL	0.009 ± 0.004	< MDL–0.267	7.8
PCB-118	0.055	0.088 ± 0.015	< MDL–0.881	66.0
PCB-138	0.279	0.390 ± 0.047	< MDL–2.330	80.6
PCB-153	0.258	0.395 ± 0.044	< MDL–2.375	95.1
PCB-158	< MDL	0.007 ± 0.005	< MDL–0.484	7.8
PCB-167	< MDL	0.004 ± 0.002	< MDL–0.134	4.9
PCB-170	< MDL	0.091 ± 0.028	< MDL–2.600	35.0
PCB-180	0.157	0.408 ± 0.064	< MDL–2.948	58.3
PCB-194	< MDL	0.022 ± 0.007	< MDL–0.399	10.7
Total PCBs	1.864	2.808 ± 0.293	0–14.911	99.0*^a^*
∑PCBs*^b^*	0.830	1.415 ± 0.164	0–9.206	97.1*^a^*
TEQ (ppt)	0.007	0.033 ± 0.007	0–0.558	75.7*^a^*

a For total PCBs, ∑PCBs, and TEQ, values are the percentage of samples with a reportable result, not percentage below MDL, because MDL is determined only for individual congeners.

b Sum of IUPAC PCB congeners 105, 118, 138, 153, 158, 167, 170, 180, 194.

**Table 3:** Multiple regression analysis of log-transformed CBT value on background variables in Mohawk men (*n* = 63) and women (*n* = 40), Akwesasne, 1992–1995.

Background variable	β-Value	SE (β)	*p*-Value
Cigarette smoking in past 2 years (yes/no)	0.360	0.136	0.009
Sex (men/women)	0.303	0.139	0.032
Oral contraceptive use in past 6 months (yes/no)*^a^*	−0.697	0.234	0.006

β, regression coefficient.

a Women only (information on oral contraceptive use was missing for five women).

**Table 4:** Multiple regression analysis of log-transformed CBT value*^a^* on serum PCB concentrations (wet weight and lipid adjusted) in Mohawk men and women, Akwesasne, 1992–1995.

	Wet weight (*n* = 103)				Lipid adjusted (*n* = 46)						
Congener	β	SE (β)	*p*-Value	β	SE (β)	*p*-Value
PCB-118	0.030	0.045	0.511	0.185	0.106	0.090
PCB-138	0.017	0.034	0.624	0.215	0.106	0.048
PCB-153	0.145	0.071	0.045	0.346	0.130	0.011
PCB-170	0.084	0.047	0.079	0.324	0.120	0.010
PCB-180	0.080	0.046	0.086	0.207	0.075	0.009
Total PCBs	0.061	0.059	0.306	0.221	0.119	0.070
∑PCBs*^b^*	0.116	0.059	0.052	0.356	0.112	0.003
TEQ	0.054	0.032	0.091	0.154	0.068	0.028

β, regression coefficient.

a Adjusted for cigarette smoking in the past 2 years and sex.

b Sum of PCB congeners 105, 118, 138, 153, 158, 167, 170, 180, and 194.
